# In Vivo Imaging of Local Gene Expression Induced by Magnetic Hyperthermia

**DOI:** 10.3390/genes8020061

**Published:** 2017-02-08

**Authors:** Olivier Sandre, Coralie Genevois, Eneko Garaio, Laurent Adumeau, Stéphane Mornet, Franck Couillaud

**Affiliations:** 1 Laboratory of Organic Polymer Chemistry, LCPO, UMR 5629 CNRS, University of Bordeaux, Bordeaux-INP, Pessac 33600, France; 2 Molecular Imaging and Innovative Therapies in Oncology, IMOTION, EA 7435, University of Bordeaux, 146 rue Léo Saignat, case 127, Bordeaux cedex 33076, France; coralie.genevois@u-bordeaux.fr; 3 Department of Electricity and Electronics, University of the Basque Country (UPV/EHU), P.K. 644, Leioa 48940, Spain; eneko.garayo@ehu.eus; 4 Institute for Condensed Matter Chemistry of Bordeaux, ICMCB, UPR 9048, CNRS, University of Bordeaux, Pessac F-33600 France; laurent.adumeau@gmail.com (L.A.); stephane.mornet@icmcb.cnrs.fr (S.M.)

**Keywords:** magnetic hyperthermia, gene therapies, heat shock protein promoter, in vivo optical imaging, magnetic polymer-coated nanoparticles

## Abstract

The present work aims to demonstrate that colloidal dispersions of magnetic iron oxide nanoparticles stabilized with dextran macromolecules placed in an alternating magnetic field can not only produce heat, but also that these particles could be used in vivo for local and noninvasive deposition of a thermal dose sufficient to trigger thermo-induced gene expression. Iron oxide nanoparticles were first characterized in vitro on a bio-inspired setup, and then they were assayed in vivo using a transgenic mouse strain expressing the luciferase reporter gene under transcriptional control of a thermosensitive promoter. Iron oxide nanoparticles dispersions were applied topically on the mouse skin or injected subcutaneously with Matrigel™ to generate so-called pseudotumors. Temperature was monitored continuously with a feedback loop to control the power of the magnetic field generator and to avoid overheating. Thermo-induced luciferase expression was followed by bioluminescence imaging 6 h after heating. We showed that dextran-coated magnetic iron oxide nanoparticle dispersions were able to induce in vivo mild hyperthermia compatible with thermo-induced gene expression in surrounding tissues and without impairing cell viability. These data open new therapeutic perspectives for using mild magnetic hyperthermia as noninvasive modulation of tumor microenvironment by local thermo-induced gene expression or drug release.

## 1. Introduction

Gene therapies are promising techniques for curing diseases either by repairing or replacing a defective gene or by expressing some therapeutic proteins or regulatory noncoding RNA. In spite of some well-known successes, such as therapy of crosslinked severe combined immunodeficiency (X-SCID) [1] and quite a lot of clinical trials [2], gene therapies are not widely used in clinical practices. Safe, specific, and efficient delivery of a therapeutic gene to an identified cell or organ still remains an important challenge but efficient control of transgene expression also requires considerable improvement.

Tight spatiotemporal regulation of gene expression, in the region where therapy is necessary and for the duration required to achieve a therapeutic effect, is very important for clinical applications of gene therapy and for minimizing systemic toxicity. Temporal control of gene expression may be achieved by several externally controlled, inducible gene promoters which respond to antibiotics [3] and other small molecules [4]. Spatial control is more frequently envisaged by using tissue-specific or disease-specific promoters, yet lacking features for both temporal and external controls. Furthermore, these specific promoters often exhibit a low level of therapeutic gene expression.

Hyperthermia in combination with the temperature-sensitive 70 kDa heat-shock protein (*HSP70*) promoter presents a unique approach, allowing noninvasive spatiotemporal control of transgene expression. We already demonstrated in vivo, by using transgenic mice and genetically modified cells, that focused-ultrasound (FUS) combined with real-time monitoring of the local temperature distribution by phase magnetic resonance imaging allows for a fine control of temperature increase and thus a good spatiotemporal control of local transgene expression by using thermosensitive promoters [5,6,7].

Magneto-activatable thermogenic nanoparticles (MTN) as heat sources also appeared as an attractive alternative, especially for deep-seated and poorly accessible tumors [8,9]. MTN injection into tumors and their subsequent heating using an alternating magnetic field has been developed as a cancer treatment for several decades, and after a phase II clinical trial at the end of 2011, it was authorized for use against severe brain cancer (glioblastoma) in combination with conventional radiotherapy and is currently proposed as a clinical tool. Depending on the temperature increase and the duration, tumor cell killing or increased susceptibility to concomitant radio- or chemotherapy has been observed [8,9,10]. MTN-based hyperthermia treatment also increases tumor sensitivity to natural killer cell-mediated lysis [11] and tumor-specific immune responses resulting from thermo-induction of heat-shock proteins (HSP) expression [12,13].

The present paper aims to demonstrate that MTNs could be used in vivo for local and noninvasive deposition of a sufficient thermal dose to trigger transgene expression by a thermosensitive promoter. Chosen MTNs are colloidal dispersions of magnetic iron oxide nanoparticles stabilized with dextran macromolecules. We first establish MTN heating properties on a bio-inspired in vitro setup, and then we move on to a thermosensitive transgenic mouse strain already characterized in vivo by bioluminescence imaging for its response to mild hyperthermia [14,15].

## 2. Materials and Methods

### 2.1. Animals and Animals Handling

Animal experiments were performed in agreement with European directives and approved by the local ethical comity (CEEA 50) under agreement A50120195. The double transgenic mice Hspa1b-LucF (+/+) Hspa1b-mPlum (+/+) [15] were housed at the University of Bordeaux facilities and maintained under 12 h light/dark cycle with water and food ad libitum. Animals were anesthetized with 2% isoflurane (Belamont, Nicholas Piramal Limited, London, UK) in air. Prior to experiments, mice were shaved with clippers and a depilatory cream was applied.

### 2.2. MTN Synthesis and Characterization

Magnetic nanoparticles (MNPs) synthesized by various chemical routes are studied for their potential use in medicine, in particular for magnetic hyperthermia [16,17]. In this work, MNPs were synthesized by alkaline coprecipitation of ferrous and ferric salts followed by a size-sorting procedure as previously described [18]. Briefly, magnetite (Fe_3_O_4_) MNPs, right after the coprecipitation reaction, were totally oxidized into maghemite (γ-Fe_2_O_3_) by boiling ferric nitrate [19] and dispersed in dilute nitric acid. Then, they were submitted to a size-sorting procedure based on the liquid–liquid phase-separation obtained by screening the electrostatic repulsions with an excess of electrolyte (HNO_3_) concentration, yet below 0.4 M (pH > 0.4) in order to prevent MNP dissolution into ions. The principle of this sorting is that the concentrated phase is enriched with the larger MNPs, whereas the dilute phase contains the smaller MNPs [18,20,21]. After repeating several phase separations and washing steps (three cycles), the sample named C1C2C3 originated from the concentrated phase fractions corresponding to the concentrated phases obtained by adding HNO_3_ electrolyte to the preceding pelleted fractions. The ions in excess were washed off with acetone and diethyl ether, resulting into a suspension in diluted HNO_3_ at pH ∼ 2 and at an iron oxide concentration of 110 g/L. These particles displayed a mean nanoparticle size of 12.4 nm (with standard deviation σ = 6.0 nm), as measured by automated particle counting on a transmission electronic microscope (TEM) image (Figure S1) This “ionic ferrofluid” was then coated by dextran T70 (Mw 70 kDa, Sigma-Aldrich, Saint-Quentin Fallavier, France) in order to be dispersible in aqueous buffer at neutral pH and also in a protein hydrogel matrix (called hereinafter Matrigel™). For this coating, a volume of 5 mL of ferrofluid at 110 g/L was added to 30 mL of dextran solution at 100 g/L and then incubated overnight under gentle shaking. This dispersion was precipitated in 120 mL of ethanol, and particles were separated magnetically from free (un-adsorbed) dextran macromolecular chains. This dextran precipitation not only allows removal of most of the polymer excess but also promotes the adhesion of glucose monomers onto the nanoparticle surface through hydrogen bonds. The pellet was dispersed in 40 mL of sterile ultrapure water (18 MΩ·cm) produced by using a SG-Labostar (7 TWF-UV system from Odemi, Grisy, France) and heated at 70 °C under vacuum in order to remove residual ethanol. Finally, free dextran chains were removed by several washes (two times with a dilution factor of about 10) with ultrapure water by tangential flow filtration (TFF) using 300 kDa cut-off ultrafiltration filters (Merck Millipore). At the end of the procedure, these magnetic nanoparticles contained 15.4% of dextran, determined by thermogravimetric analysis (TGA) (Figure S2). Finally, the so-prepared ferrofluid, thereafter denominated C1C2C3@dex, was concentrated by TFF in phosphate-buffered saline (PBS) to obtain an iron oxide concentration of 117 g/L as measured by a UV–vis spectrum (Figure S3). Their hydrodynamic diameter was measured by dynamic light scattering (DLS) after 200-times dilution in water with a Malvern™ NanoZS apparatus operating at 90° scattering angle (Figure S4), giving a Z-average diameter of 40.2 nm with low polydispersity (PDI = 0.215). Their specific heating power was characterized by the “specific absorption rate” (SAR) in the alternating magnetic field (AMF) conditions used (*H* = 10.2 kA/m at 755 kHz). A value for specific absorption rate (SAR) = 94 ± 1 W/g was found from the initial slope of the temperature profile within the first 3 s of AMF application (Figure S5). In previous work containing extensive magnetic characterization, we showed that this sample has a SAR verifying the linear response theory based on Néel and Brown relaxation processes of the magnetic moments, using independent magnetic measurements of the specific magnetization and of the magnetic anisotropy constant [18].

### 2.3. In Vitro MTN Experiments (Phantoms)

**Figure 1:**
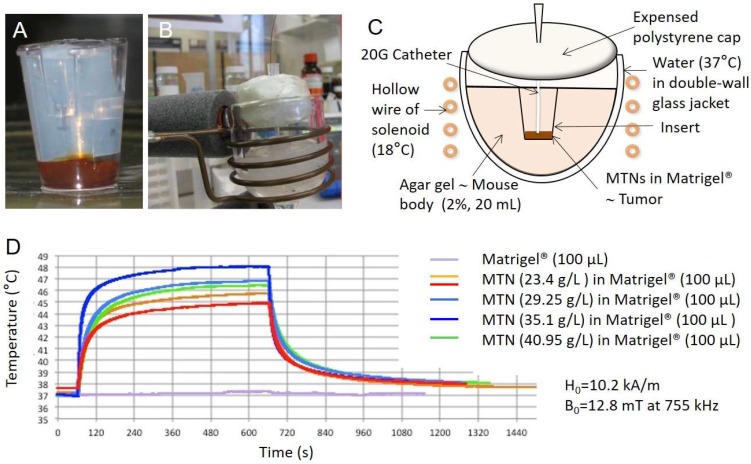
Heating performances of MTNs in a bio-inspired phantom. (**A**) Phantoms were made of 100 µL Matrigel™ containing magneto-activatable thermogenic nanoparticles (MTNs) at different concentrations and poured into a cell culture insert (24 wells; ø pores: 0.8 µm) to mimic a tumor; (**B**) Insert was covered by an agar plug and inserted into a 20 mL agar gel (2% w/v) of thermal inertia equivalent to the mouse body. Phantom was placed into a thermostatic chamber maintained at 37 °C through water circulation within double-walled glass water jacket to represent “active” thermal regulation by the mouse blood circulation, and located in the middle of the solenoid. The complete system reached thermal equilibrium at 37 °C before the alternating magnetic field was generated. An optical fiber thermal probe (Opsens® OTG-M420) was introduced into the insert up to reaching the MTN/Matrigel™ mixture through a catheter (20 G, TERUMO®); (**C**) Schematic representation of the experimental setup; (**D**) Time courses of temperature in the magnetic field of induction *B* = 12.8 mT at 755 kHz (*H* = 10.2 kA/m) according to different MTN concentrations.

An aliquot of C1C2C3@dex nanoparticles (20, 25, 30, or 35 µL), diluted to 50 µL with water, was mixed with 50 µL of Matrigel™ (BD Matrigel™ Basement Membrane Matrix; Becton, Dickinson and Company, San José, CA, USA) at a temperature near 4 °C. Previously, it was checked that Matrigel™ diluted twice still jellified when raising the temperature to 37 °C. The mixture was poured into a cell culture insert (24 wells; ø pores: 0.8 µm) and covered by an agar plug (Figure 1A). The insert was placed into an agar gel (20 mL; 2% w/v) modeling the heat capacity of a mouse (body weight around 20 g) which was placed in a thermostatic double-wall chamber (Figure 1B). The system was then placed in the middle of the solenoid and maintained at 37 °C (Figure 1C) by the water circuit linked to a regulating bath (Huber Polystat CC, Offenburg, Germany) before the alternating magnetic field (AMF) was generated by the coil. An optical fiber thermo-probe (OTG-M420; Opsens™ Inc., Québec, QC, Canada) was introduced inside the insert through a catheter (20 G, TERUMO®, Terumo Medical Corporation, Somerset, NJ, USA) to reach the C1C2C3@dex nanoparticle/Matrigel™ mixture (Figure 1C).

### 2.4. In Vivo MTN Experiments (Animals)

MTNs were diluted in water and applied topically on the mouse skin. Diluted MTNs were also mixed with Matrigel™ at 4 °C and injected subcutaneously on the back of the mouse to generate so-called “pseudotumor”. After 30 min, the Matrigel™ was gelled and the mouse was placed inside the solenoid. Optical fiber thermo-probe (OTG-M420; Opsens™, Québec, QC, Canada) was inserted into the pseudotumor using a Teflon™ catheter (20 G, TERUMO®) as guide. Room temperature around the coil was monitored during the experiment and maintained at 20 °C.

### 2.5. Hyperthermia Setup and Protocol

The electric generator (SEIT ELECTRONICA, Junior™ 3.5 kW model creates an alternating magnetic field (AMF) inside a 4-turn copper-ring solenoid of 55 mm outer diameter, 48 mm inner diameter, and 38 mm height. The solenoid is refrigerated by a cold (18 °C) water flux inside the 3.5 mm diameter, 0.4 mm wall thick, hollow wires of the solenoid. Magnetic field intensity is *H* = 10.2 kA/m, induction *B* = 12.8 mT and frequency 755 kHz, as determined by finite element modeling for magnetics (FEMM, http://www.femm.info), after measuring the high voltage (747 V) and deducing the current (234 Amps) from the calculated coil impedance (*R* = 3.2 Ω) and inductance (*L* = 4.5 × 10^−9^ H). The field value calculated by finite element modeling was also checked experimentally by measuring the electromotive force in a scout coil (1 turn of 17.5 mm diameter): 29.4 V peak to peak.

Homemade software was developed in LabVIEW™ allowing temperature regulation during the heating process according to predefined parameters (i.e., duration (in seconds), maximum temperature, and thermal dose above a threshold temperature). The thermal dose is defined as the integral of the temperature vs. time profile above a predetermined threshold, usually 42 °C. This definition is equivalent to the “equivalent total time at 43 °C” expressed in “degree-minute” (tdm_43_) [22].

### 2.6. In Vivo Bioluminescence Imaging (BLI)

BLI was performed at the Vivoptic platform (Bordeaux University & CNRS, UMS 3767) using a NightOWL II LB 983 calibrated system equipped with an NC 100 CCD deep-cooled camera (Berthold Technologies™, Bad Wildbad, Germany). Mice were injected intraperitoneally with d-luciferin (2.9 mg in 100 µL PBS, Promega™, Madison, WI, USA,) and sedated 5 min later. Bioluminescence images (1 min exposure, 4 × 4 binning) and photographs (100 ms exposure) were taken 8 min after the luciferin injection. A low light emitting standard (Glowell, Lux Biotechnology Limited, Edinburgh, UK) was placed next to the animal during each image acquisition to provide a quality control. Pseudocolor images representing the spatial distribution of emitted photons were generated using IndiGO 2 software (Berthold Technologies™) and superposed to the corresponding photographs.

## 3. Results

### 3.1. Heating Performances of MTN In Vitro on Bio-Inspired Phantom

We first assayed MTN heating performances using an in vitro bio-inspired setup. MTNs were mixed with Matrigel™, a natural component of the tumor microenvironment, and the mixture was placed into a 20 mL agar gel to mimic a mouse weight of about 20 g. The tumor phantom model was designed with a volume of 100 µL in order to exceed the diameter of 1.1 mm proposed by Rabin as a minimum size of magnetic sample to exhibit macroscopic heating compared to environmental temperature [23]. The agar gel was actively thermo-regulated at 37 °C using a water flux. MTNs were mixed with Matrigel™ at a final iron oxide concentration of 23.4, 35.1, and 40.8 g/L. The maximum temperature that can be reached in Matrigel™ phantoms depends on their volume and on the concentration of their contained MTNs. Figure 1D shows the temperature profiles obtained for the maximum power of the generator providing magnetic field intensity of *B* = 12.8 mT at 755 kHz. The temperature, initially at 37 °C, rapidly increases and reaches a plateau that is maintained as long as the magnetic field is maintained, corresponding to a perfect balance between the heat flux created by the MTNs and the thermal losses at the interface between the magnetic phantom and the surrounding hydrogel mimicking the mouse body. Activation was repeated several times in order to check the reproducibility of the biomimetic system. Observation of the MTN-containing Matrigel™ after the experiment reveals a poor homogeneity of the spatial distribution of MTNs for an iron oxide concentration of 40.95 g/L. At a concentration of 35.1 g/L, the maximum temperature obtained is 48 °C while MTNs are homogeneously distributed in the Matrigel™ tumor phantom.

### 3.2. In Silico Modeling of Temperature Distribution in the Bio-Inspired Phantom

**Figure 2:**
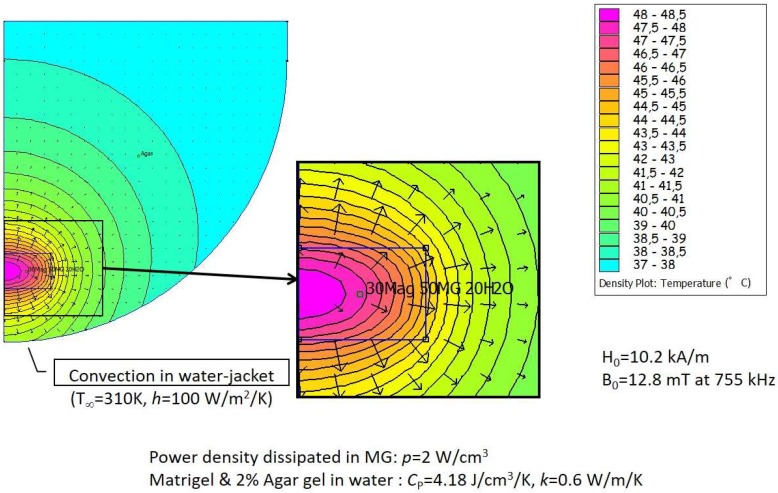
Numerical modeling of thermal distribution in the Matrigel™ phantom with 3709 nodes using finite element modeling for magnetics (FEMM) freeware (http://www.femm.info/). The power density was chosen to correspond to an MTN concentration of 35.1 g/L under an alternating magnetic field *B* = 12.8 mT at 755 kHz (*H* = 10.2 kA/m). At thermal equilibrium (stationary state of the heat equation), maximal temperatures of 48 °C in the center of the Matrigel™ insert and of 44 °C at the insert periphery were predicted by the numerical model.

Numerical modeling of thermal distribution was used to predict MTN heating upon activation. The finite element software FEMM can perform numerical simulations not only of magnetics but also of the Fourier heat diffusion and convection equation. The grid model took into account all parameters of the experimental device, including dimensions of the various compartments, fixed temperature (37 °C) at the boundary of the glass jacket with convective conditions, and thermal characteristics of the Matrigel™ and agar media such as heat capacity and conductivity, taken as pure water values (*C*_P_ = 4.18 J/cm^3^/K, *k* = 0.6 W/m/K). Figure 2 shows the modeling results for MTN concentration of 35 g/L in the 100 µL Matrigel™ phantom. The iron oxide mass in the phantom (3.5 mg) can irradiate a power 3.5 × 10^−3^ × 94 = 0.33 W in a 100 µL volume, thus a power density of 3.3 W/cm^3^. A somehow lower estimated value *p* = 2 W/cm^3^ was taken in the simulation to take into account the decrease of SAR when the MNPs are partially blocked within the gel matrix as compared to the homogeneous fluid (Brown relaxation mechanism is likely impeded). This 40% decrease of SAR is arbitrary, but it is quite in line with a systematic study of the local concentration’s negative effect on SAR for similar hydrophilic polymer-coated MTNs [24]. The maximum temperature is predicted in the center of Matrigel™ insert, and temperature gradually decreases with distance from the center. The temperature difference is nearly 10 ° C between the Matrigel™ center and the border of the agar block containing the insert, yet with variation between 45 °C and 47 °C depending on the axial and lateral coordinates. The temperature predicted by the numerical model in the center rose to 48.5 °C, which is very close to the experimental value of 48 °C reported in Figure 1D for the same concentration (35 g/L). However, these curves show non-monotonous variation with concentration in the experiments, which might be ascribed to Matrigel™ heterogeneity or unprecise localization of the optical fiber temperature probe (in agreement with the thermal gradient calculated numerically).

### 3.3. Topical Application of MTN Solution on Transgenic Mouse Skin

**Figure 3:**
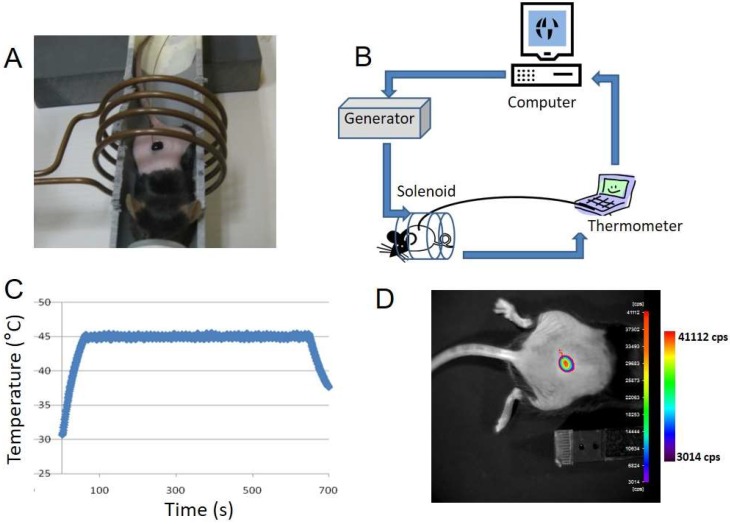
Imaging of heat-induced expression of luciferase by magnetic activation of MTN applied on the transgenic mouse skin. (**A**) An MTN drop (69 g/L, 100 µL water) was applied on *HSP70*–LucF transgenic mouse skin. The mouse was placed on a bed aligned with the central axis of the solenoid. An optical fiber thermoprobe was immersed in the MTN droplet; (**B**) On/off cycles of alternating magnetic field (*B* = 12.8 mT at 755 kHz (*H* = 10.2 kA/m)) were created by a computer-controlled generator according to the temperature recorded in the drop (T_on_ = 44°C, T_off_ = 45°C); (**C**) Examples of temperature measurements in the MTN solution; (**D**) Bioluminescence imaging of thermo-induced luciferase expression by the mouse skin, 6 h after magnetic hyperthermia.

MTN aqueous suspension (69 g/L, 100 µL) was applied on the skin of *HSP70*–LucF transgenic mouse. Mouse was placed in the middle of the solenoid and an optical fiber thermoprobe was immersed in the MTN fluid droplet (Figure 3A). Temperature was monitored using LabVIEW™ homemade program to drive the generator and thus the magnetic field created by the solenoid (Figure 3B). The magnetic field generator was switched off when the temperature inside the MTN drop reached 45 °C and was switched on again at 44 °C, leading to a saw tooth temperature profile. Applied magnetic field was *B* = 12.8 mT at 755 kHz (*H* = 10.2 kA/m). As shown in Figure 3C, heating was maintained for 10 min until the AMF was definitively switched off, with temperature decreasing rapidly to the baseline skin temperature (32 °C). BLI measurement was performed 6 h after magnetic heating according to previous data reporting *HSP70*-dependent LucF expression [15]. Figure 3D illustrates *HSP70*-dependent LucF expression as revealed by BLI. As controls, MTNs were applied on the mouse skin without magnetic field or the mouse was placed into the magnetic field for 10 min without MTNs; in these cases, no BLI signal was detected [25].

### 3.4. Subcutaneous MTN-Containing Matrigel™ Pseudotumors in Mice

**Figure 4:**
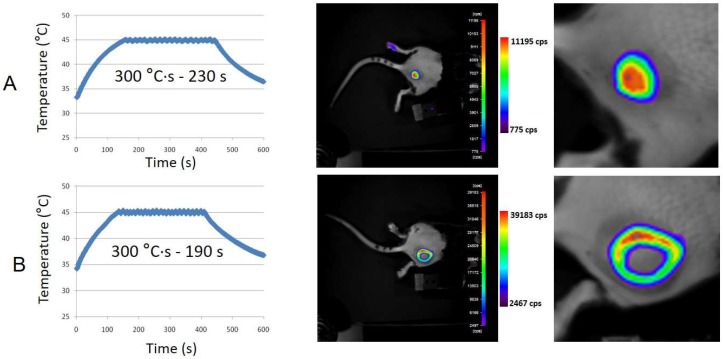
Imaging of heat-induced expression of luciferase by magnetic activation of Matrigel™ pseudotumors containing MTNs. Cold Matrigel™ (50 µL) added to MTNs (30 µL at 115 g/L, 20 µL water) leading to 100 µL at 34.5 g/L final MTN concentration were injected subcutaneously and allowed to solidify for 30 min at mouse body-temperature. The mouse is placed in the middle of the solenoid. An optical fiber thermoprobe (360 µm) was inserted in the Matrigel™ volume using a Teflon™ catheter. Magnetic field (*B* = 12.8 mT at 755 kHz (*H* = 10.2 kA/m)) is created by a PC-controlled generator (on/off cycles) according to temperature recorded in the Matrigel™ volume. Predefined thermal dose was 300 °C×s calculated above 42 °C, or an equivalent time at 43 °C, tdm_43_ = 5 min, and upper limit temperature was set at 45 °C. Line (**A**) and (**B**) are BLI images of thermo-induced luciferase expression by the mouse tissues, 6 h after magnetic hyperthermia.

To test MTN-induced intratumoral hyperthermia with further realism towards therapeutic application, Matrigel™ pseudotumors containing MTNs (35.1 g/L) were created subcutaneously in HSP/LucF transgenic mice by injection of the mixture in the cold fluid state. A Teflon™ catheter was introduced into the pseudotumors to be used as a guide for the fiber optics temperature probe. Temperature was monitored using software developed under LabVIEW™ to drive the generator and thus the magnetic field inside the solenoid. Maximum temperature was fixed at 45 °C and running time was defined according to calculation of the predefined thermal dose expressed as tdm_43_ (i.e., time interval equivalent to a thermal dose calculated above a threshold temperature of 42 °C). As shown in Figure 4, magnetic activation of Matrigel™ pseudotumors containing MTNs, heating results in LucF expression by mouse tissues as detected by BLI 6 h after heating. Different types of BLI patterns were obtained. Some mice exhibited a “Gaussian type” BLI pattern (Figure 4A; *n* = 5) whereas some other exhibited “ring shape” BLI patterns with no BLI signal in the middle of the heated zone (Figure 4B; *n* = 13). The pattern was not related to the predefined thermal dose, as illustrated in Figure 4.

Soaking the leg of the transgenic mice in warm water (45 °C, 8 min) induces *HSP70* promoter activation and LucF expression, as detected by BLI 6 h later [14,15]. To determine the physiological status of the central area of the ring-shaped pattern, the mouse paw was immersed in a water bath (45 °C, 8 min, tdm_43_ = 24 min). Six hours after this heating, the whole paw expresses luciferase except the central area of the ring. This lack of signal therefore corresponds to an area where cells have lost the ability to express luciferase. Transgenic mice with Matrigel™ pseudotumors containing MTNs without AMF application or with Matrigel™ pseudotumors alone and placed in the AMF did not exhibit BLI signal. Mice bearing pseudotumors with MTN concentrations lower than 35 g/L did not exhibit BLI signal (11 g/L, 15 min AMF on; 15 g/L 15 min AMF on; 18 g/L 15 min AMF on; *n* = 5; [25]).

### 3.5. In Silico Modeling of Temperature Distribution in MTN-Containing Pseudotumors

**Figure 5:**
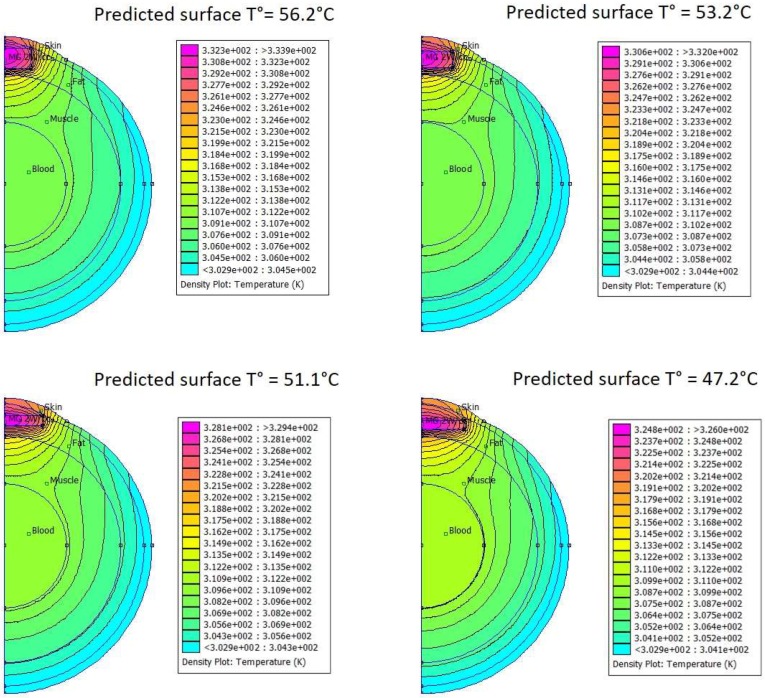
Numerical modeling (FEMM grid with ~7800 nodes) of temperature spatial distribution in and around a subcutaneous pseudotumor placed in the fat layer (3 mm thick) below skin (1 mm) and above muscles for different tumor shapes at constant 100 µL volume. Blood volume is 2.1 mL. MTNs are supposed to dissipate a heat power density *p* = 2 W/cm^3^ under an alternating magnetic field of *B* = 12.8 mT at 755 kHz (*H* = 10.2 kA/m). Thermal parameters (heat capacity and conductivity) used are taken from literature [22]: (*C*_P_ = 3.28 J/cm^3^/K; *k* = 0.37 W/m/K) for skin, (*C*_P_ = 1.93 J/cm^3^/K; *k* = 0.16 W/m/K) for fat, (*C*_P_ = 3.87 J/cm^3^/K; *k* = 0.48 W/m/K) for muscle, (*C*_P_ = 4.18 J/cm^3^/K; *k* = 0.6 W/m/K) for blood pool, supposed isothermal at T_0_ = 310 K. Predicted temperatures at the pseudotumor surface were 56.2, 53.2, 51.1, and 47.2 °C according to pseudotumor cylindrical shape heights of 2.6, 2, 1.34, and 1.04 mm, respectively (for the same 100 µL volume).

Using the same finite element modeling FEMM software as was used for bio-inspired phantoms, temperature-mapping in MTN-containing Matrigel™ pseudotumors was assayed upon activation with different tumor shapes that can arise after subcutaneous injection of a given volume of 100 µL. Thermal characteristics of the different tissues were obtained from the literature [26]. Figure 5 shows the modeling results for MTN concentration of 35 g/L in 100 µL Matrigel™ pseudotumors located below the skin. According to the pseudotumor shape, the estimated temperature on the skin surface facing the BLI camera increased from 46.2 °C (flat disk-like tumor) up to 57.2 °C (cylindrical tumor of larger thickness, thus lower surface area and less thermal loss to the surrounding tissues).

## 4. Discussion

In the present paper, we demonstrate the feasibility of inducing gene expression in vivo by magnetic hyperthermia. When MTNs were placed in an alternating magnetic field, they produced heat which dissipated into the surrounding environment. The challenge was to use MTN-based heating in physiological environment and to generate enough thermal energy to activate the thermosensitive promoter in cells of surrounding tissues. The transgenic mouse used is expressing the reporter gene *LucF* under transcriptional control of the *HSP70* thermosensitive promoter, and this mouse has been already fully characterized for its response to mild hyperthermia [14]. For temperature above 43 °C, temperature-induced *HSP70* promoter activation was modulated by both temperature as well as duration of hyperthermia and the reporter gene expression can be modeled by an Arrhenius analysis [14]. Typically, by soaking a mouse paw in a water bath at 45 °C for 8 min (calculated thermal dose = 1440 °C·s above 42 °C, thus tmd_43_ = 24 min), a high BLI signal was expressed by skin [14,15]. The calculated average activation energy required for activation of *HSP70* promoter in this mouse was 559 kJ/mol leading to a K-factor of about 2, indicating that for temperatures above 43 °C, an increase in temperature of 1 °C is equivalent to decreasing the exposure time by a factor 2 [14]. The light emission after heating attests for both *HSP70* promoter activation and cell viability. The BLI readout indeed requires *LucF* translation and both oxygen and ATP-dependent LucF enzymatic activity.

To mimic the thermic conditions in mice exposed to magnetic hyperthermia, we reported at first a phantom system consisting of a 20 mL soft aqueous gel, with a peripheral water-circulating system thermalized at 37 °C representing blood circulation. To mimic a tumor stroma, an MTN droplet was diluted to 100 µL with water and Matrigel™, which is a commercially available natural component of the extracellular matrix. In this medium, the state of dispersion of MTNs is believed to be closed to mouse extracellular medium (i.e., they are partially blocked in the water pocket of the tight network. The upper concentration of MTNs in Matrigel™, without impeding the gel formation, is limited to about 35 g/L, which constituted the first limitation. Higher concentration results in MTN precipitation in Matrigel™ and impairs heating performance. As a second limitation, we found that in such conditions, heating capacity of MTNs is limited, lowered by a factor of around 40% (as estimated by the heat power density *p* = 2 W/cm^3^ necessary for according experiments and thermic simulations, as compared to *p* = 3.3 W/cm^3^ calculated from the SAR in liquid state). At a temperature of 37 °C, Matrigel™ indeed forms a dense protein network of 50–500 nm mesh-size [27], in which the polysaccharide-coated MNPs presumably are adsorbed and blocked, hampering the Brown relaxation mechanism of their magnetic moment under an AMF. When Matrigel™ pseudotumors containing MTNs (35 g/L) were placed in the alternative magnetic field, the temperature rose and rapidly reached a plateau at about 48 °C in the magnetic field conditions used. Lower concentrations of MTNs resulted in lower plateau temperatures, and thus the lower limit of 43 °C required to induce cellular heat stress was not reached [28].

Application of MTNs in aqueous liquid droplets on the mouse skin and subsequent activation by alternating magnetic field resulted in significant temperature increase, despite heat dissipation in air. To prevent any hazard and extensive tissues damage, the temperature was recorded continuously and limited to 45 °C. Such protocol ensures thermal dose calculation in real time and allowed for LucF expression. Although not as extensively studied in the present work as would be needed for a quantitative relationship, the BLI signal was well correlated with the thermal dose deposited on the mouse skin, defined as the integral of time–temperature curve above 42 °C with a coefficient of determination R^2^ = 0.999 (*n* = 3: linear fit, Figure S6) and was consistent with previously reported data using warm water as heating source.

MTNs diluted in cold liquid Matrigel™ at 35 g/L and injected subcutaneously in mouse formed a solid mass when reaching physiological temperature of the animal. Placing the mouse into the alternating magnetic field resulted in MTN activation. The main result of this work was evidence that magnetically induced heat generation was sufficient to induce *HSP70-*dependent transgene expression in surrounding tissues. The temperature was recorded continuously by placing a thermal probe inside the Matrigel™ mass: Although the position of the 420 µm fiber in the millimeter-sized tumor phantom was not sufficiently precise to guarantee to be in the exact geometrical center of the pseudotumor, the temperature rise could be limited by software control of the AMF generation to 45 °C. However, certainly due to this imprecision of probe localization, BLI patterns of transgene activation appeared very variable and not well correlated to the recorded thermal dose. In particular, ring-shaped BLI patterns without a light-emitting area in the middle were observed in some cases. Ring-shaped activation patterns have been already reported and were resulting in local overheating, leading to central tissues necrosis and *HSP70* activation at the periphery [6]. This suggest that the recorded temperature was not representative of the overall temperature rise in the Matrigel™ mass and that the thermal probe may be not located in the warmer point of the Matrigel™ pseudotumor volume, but rather at its periphery, explaining the underestimation of the recorded temperature (and hence overheating and cell catabolism in the central part).

Modeling thermal exchanges by finite element modeling (FEM) simulation of the heat equation with biologically relevant parameters provided good correlation between simulations and experimental measurements. It is indeed the case that, when applied to the phantom setup, FEM provided consistent prediction of the maximum temperature reached at thermal equilibrium (i.e., when generated heat is balanced by thermal losses). For in vivo experiments using MTNs dispersed in Matrigel™, numerical modeling clearly illustrated that the temperature field is not homogeneous in the Matrigel™ volume. The higher temperature was calculated in the geometrical center of the Matrigel™ volume, and the predicted temperature profiles exhibited a centrifuge gradient. In vivo, perfect positioning of the thermal probe in the center of the Matrigel™ could neither be precisely achieved nor checked. Incorrect positioning provided false evaluation of the overall temperature in the Matrigel™ and further mistaking of the feedback loop software controlling the magnetic field. This may explain in part the ring-shaped BLI patterns ascribed to overheating and catabolism/necrosis of the cells in contact with the hottest spots. Modeling also illustrated changes in thermal profiles according to pseudotumor shapes. MTN/Matrigel™ mixtures were injected in a fluid state and solidified in various shapes, ranging from “almost spherical” to “flat disk”. Modeling prediction revealed up to 9 °C of difference between expected temperatures on pseudotumor surface according to the different shapes, as ascribed to different surface-to-volume ratio and thus thermal losses to surrounding matrix, resulting in huge differences in the thermal dose delivered to tissues.

The *HSP70* promoter is activated not only by heat stress but also in response to a variety of stresses of both environmental and physiological origins [29]. Stressors include oxidative stress [30], toxic compounds [29], hypoxia, ischemia, acidosis, energy depletion, cytokines, and UV radiation [31]. In the current study, no BLI signal was detected after MTN topical application or MTN pseudotumor injection in absence of magnetic field activation. It is also noted that only female mice were used for the study to avoid BLI resulting from mechanical stresses and resulting scarring ascribed to fighting among males. In literature, it has been shown that intracellular magnetic field-activated MTN can activate the *HSP70* response in vitro [13] or induce toxicity on targeted cancer cells without a detectable temperature rise [10]. Intracellular internalization of MTNs is not expected to occur in our current experiments, as MTN suspension was deposited on skin for a very short time or the MTNs were embedded in Matrigel™. Activation of the *HSP70* response without consistent temperature increase was not observed. For instance, when recorded temperature did not reach a temperature above 43 °C or the thermal dose required for heat-induced HSP70 activation by using lower MTN concentration in Matrigel™ pseudotumors or shorter activation times, no BLI signal was detected. In other words, a macroscopic temperature rise was necessary to induce *HSP70* gene expression in this in vivo study.

The current work clearly revealed current practical limitations of the MTN-based strategy for controlling gene expression. As stated above, concentrations of the MTN required for *HSP70* promoter activation is very high, close to the limit of dispersibility of the MNPs in biological media. More efficient MNPs (exhibiting higher SAR) may be necessary to develop for future studies. Difficulties also occurred in controlling MTN homogeneity, which induced variations in the temperature field distribution. Finally, the major limitation is due to inadequate methods for temperature control in vivo and for determination of MTN distribution within the tumor microenvironment, resulting in unexpected events such as overheating. Possible amelioration could be to perform magnetic resonance imaging of the pseudotumor right before the AMF application, in order to check the exact position of the catheter and the fiber optics temperature probe.

## 5. Conclusions

In conclusion, we showed that polysaccharide-coated maghemite nanoparticles either deposited on the skin or injected into subcutaneous tumor phantoms were able to induce in vivo mild hyperthermia compatible with thermo-induced gene expression in surrounding tissues, without impairing cell viability. Although limitations still remain for finer temperature measurement and limitation, and thus, control of transgene expression, these data opened new therapeutic perspectives for using mild magnetic hyperthermia for noninvasive modulation of the tumor microenvironment by local thermo-induced gene expression or local drug release.

## Supplementary Materials

The following are available online at www.mdpi.com/2073-4425/8/2/61/s1, Figure S1: Transmission electron microscopy (TEM) image acquired at 80 kV of C1C2C3 MNP cores; Figure S2: Thermogravimetric Analysis (TGA) curve of C1C2C3@dex MNPs; Figure S3: UV-vis absorption spectrum (Spectramax™ M2E) of C1C2C3@dex MNPs; Figure S4: Dynamic light scattering (DLS) curve (Malvern Nanosizer™) of C1C2C3@dex MNPs; Figure S5: Temperature profiles for C1C2C3@dex MNPs in drop embedded in 20 mL Agar gel to mimic the mouse body; Figure S6: Quantitative relationship between thermal dose and BLI signal for topical deposition of MTN droplet on mouse skin.

## References

[1] Cavazzana-Calvo M., Hacein-Bey S., de Saint Basile G., Gross F., Yvon E., Nusbaum P., Selz F., Hue C., Certain S., Casanova J.L. (2000). Gene therapy of human severe combined immunodeficiency (SCID)-X1 disease. Science.

[2] Edelstein M.L., Abedi M.R., Wixon J. (2007). Gene therapy clinical trials worldwide to 2007—An update. J. Gene Med..

[3] Iida A., Chen S.T., Friedmann T., Yee J.K. (1996). Inducible gene expression by retrovirus-mediated transfer of a modified tetracycline-regulated system. J. Virol..

[4] Clackson T. (1997). Controlling mammalian gene expression with small molecules. Curr. Opin. Chem. Biol..

[5] Deckers R., Quesson B., Arsaut J., Eimer S., Couillaud F., Moonen C.T. (2009). Image-guided, noninvasive, spatiotemporal control of gene expression. Proc. Natl. Acad Sci. USA.

[6] Eker O.F., Quesson B., Rome C., Arsaut J., Deminière C., Moonen C.T., Grenier N., Couillaud F. (2011). Combination of cell delivery and thermoinducible transcription for in vivo spatiotemporal control of gene expression: A feasibility study. Radiology.

[7] Fortin P.-Y., Lepetit-Coiffé M., Genevois C., Debeissat C., Quesson B., Moonen C.T.W., Konsman J.P., Couillaud F. (2015). Spatiotemporal control of gene expression in bone-marrow derived cells of the tumor microenvironment induced by MRI guided focused ultrasound. Oncotarget.

[8] Thiesen B., Jordan A. (2008). Clinical applications of magnetic nanoparticles for hyperthermia. Int. J. Hyperth. Off. J. Eur. Soc. Hyperthermic Oncol. N. Am. Hyperth. Group.

[9] Kobayashi T. (2011). Cancer hyperthermia using magnetic nanoparticles. Biotechnol. J..

[10] Creixell M., Bohórquez A.C., Torres-Lugo M., Rinaldi C. (2011). EGFR-targeted magnetic nanoparticle heaters kill cancer cells without a perceptible temperature rise. ACS Nano.

[11] Kubista B., Trieb K., Blahovec H., Kotz R., Micksche M. (2002). Hyperthermia increases the susceptibility of chondro- and osteosarcoma cells to natural killer cell-mediated lysis. Anticancer Res..

[12] Ito A., Matsuoka F., Honda H., Kobayashi T. (2004). Antitumor effects of combined therapy of recombinant heat shock protein 70 and hyperthermia using magnetic nanoparticles in an experimental subcutaneous murine melanoma. Cancer Immunol. Immunother..

[13] Moros M., Ambrosone A., Stepien G., Fabozzi F., Marchesano V., Castaldi A., Tino A., de la Fuente J.M., Tortiglione C. (2015). Deciphering intracellular events triggered by mild magnetic hyperthermia in vitro and in vivo. Nanomed..

[14] Deckers R., Debeissat C., Fortin P.-Y., Moonen C.T.W., Couillaud F. (2012). Arrhenius analysis of the relationship between hyperthermia and Hsp70 promoter activation: A comparison between ex vivo and in vivo data. Int. J. Hyperth. Off. J. Eur. Soc. Hyperthermic Oncol. N. Am. Hyperth. Group.

[15] Fortin P.-Y., Genevois C., Chapolard M., Santalucía T., Planas A.M., Couillaud F. (2014). Dual-reporter in vivo imaging of transient and inducible heat-shock promoter activation. Biomed. Opt. Express.

[16] Périgo E.A., Hemery G., Sandre O., Ortega D., Garaio E., Plazaola F., Teran F.J. (2015). Fundamentals and advances in magnetic hyperthermia. Appl. Phys. Rev..

[17] Duguet E., Vasseur S., Mornet S., Devoisselle J.-M. (2006). Magnetic nanoparticles and their applications in medicine. Nanomed..

[18] Garaio E., Sandre O., Collantes J.-M., Garcia J.A., Mornet S., Plazaola F. (2015). Specific absorption rate dependence on temperature in magnetic field hyperthermia measured by dynamic hysteresis losses (ac magnetometry). Nanotechnology.

[19] Tourinho F. A., Franck R., Massart R. (1990). Aqueous ferrofluids based on manganese and cobalt ferrites. J. Mater. Sci..

[20] Massart R., Dubois E., Cabuil V., Hasmonay E. (1995). Preparation and properties of monodisperse magnetic fluids. J. Magn. Magn. Mater..

[21] Arosio P., Thévenot J., Orlando T., Orsini F., Corti M., Mariani M., Bordonali L., Innocenti C., Sangregorio C., Oliveira H. (2013). Hybrid iron oxide-copolymer micelles and vesicles as contrast agents for MRI: Impact of the nanostructure on the relaxometric properties. J. Mater. Chem. B.

[22] Sapareto S.A., Dewey W.C. (1984). Thermal dose determination in cancer therapy. Int. J. Radiat. Oncol. Biol. Phys..

[23] Rabin Y. (2002). Is intracellular hyperthermia superior to extracellular hyperthermia in the thermal sense?. Int. J. Hyperthermia.

[24] Piñeiro-Redondo Y., Bañobre-López M., Pardiñas-Blanco I., Goya G., López-Quintela M.A., Rivas J. (2011). The influence of colloidal parameters on the specific power absorption of PAA-coated magnetite nanoparticles. Nanoscale Res. Lett..

[25] Sandre O., Genvois C., Garaio E., Adumeau L., Mornet S., Couillaud F. (2017).

[26] Levy A., Dayan A., Ben-David M., Gannot I. (2010). A new thermography-based approach to early detection of cancer utilizing magnetic nanoparticles theory simulation and in vitro validation. Nanomedicine Nanotechnol. Biol. Med..

[27] Poincloux R., Lizárraga F., Chavrier P. (2009). Matrix invasion by tumour cells: A focus on MT1-MMP trafficking to invadopodia. J. Cell Sci..

[28] Van Rhoon G.C., Samaras T., Yarmolenko P.S., Dewhirst M.W., Neufeld E., Kuster N. (2013). CEM43 °C thermal dose thresholds: A potential guide for magnetic resonance radiofrequency exposure levels?. Eur. Radiol..

[29] Morimoto R.I. (1993). Cells in stress: transcriptional activation of heat shock genes. Science.

[30] Freeman M.L., Borrelli M.J., Syed K., Senisterra G., Stafford D.M., Lepock J.R. (1995). Characterization of a signal generated by oxidation of protein thiols that activates the heat shock transcription factor. J. Cell. Physiol..

[31] Kregel K.C. (2002). Heat shock proteins: Modifying factors in physiological stress responses and acquired thermotolerance. J. Appl. Physiol. Bethesda Md 1985.

