# Supplementary material for: In Vivo Imaging of Local Gene Expression Induced by Magnetic Hyperthermia
Source: Genes (Basel). 2017 Feb 8;8(2):61. doi: 10.3390/genes8020061 (PMC5333050; doi:10.3390/genes8020061)
Supplement: Supplementary file 1 [file genes-08-00061-s001.docx]

**Supplementary Materials: In Vivo Imaging of Local Gene Expression Induced by Magnetic Hyperthermia**

Olivier Sandre, Coralie Genevois, Eneko Garaio, Laurent Adumeau, Stéphane Mornet and Franck Couillaud


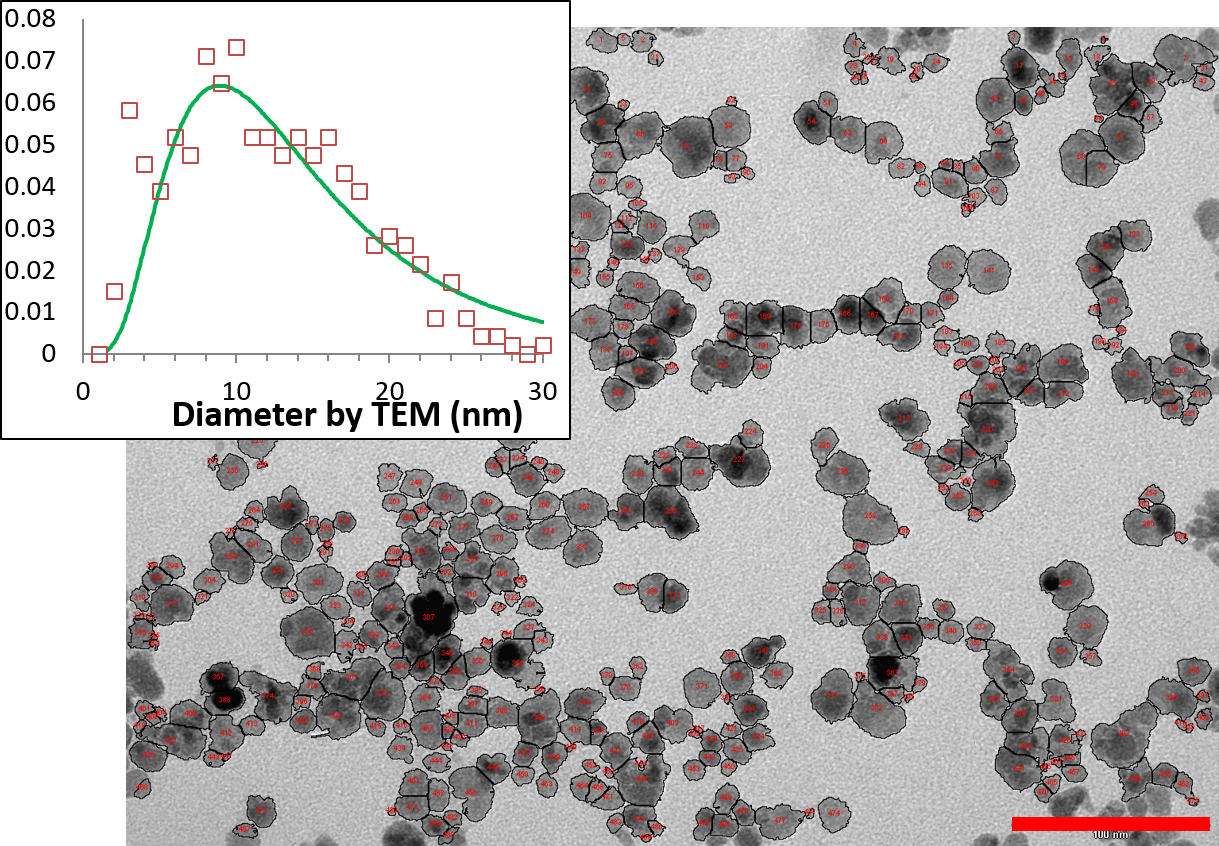


**Figure S1.** Transmission electron microscopy (TEM) image acquired at 80 kV of C1C2C3 MNP cores diluted at 80 µg/mL analyzed by automated particle counting using ImageJ (https://imagej.nih.gov/ij/). The scale bar length is 100 nm. The histogram shown in inset was built on 464 MNPs, leading to mean diameter and standard deviation: d_TEM_ = 12.4 ± 6.0 nm.


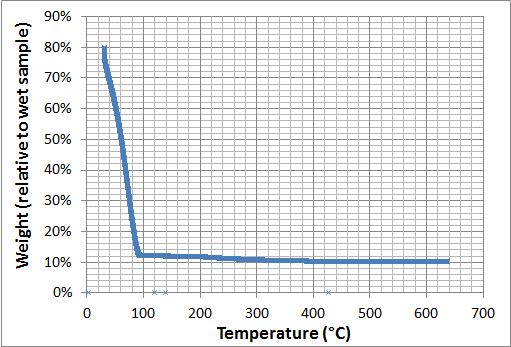


**Figure S2.** Thermogravimetric Analysis (TGA) curve of C1C2C3@dex MNPs (ramp curve in air, Q500 TA Instruments™, New Castle, DE, USA): dry matter represents 12.0% of which 1.6% is polymer, 10.4% is iron oxide.


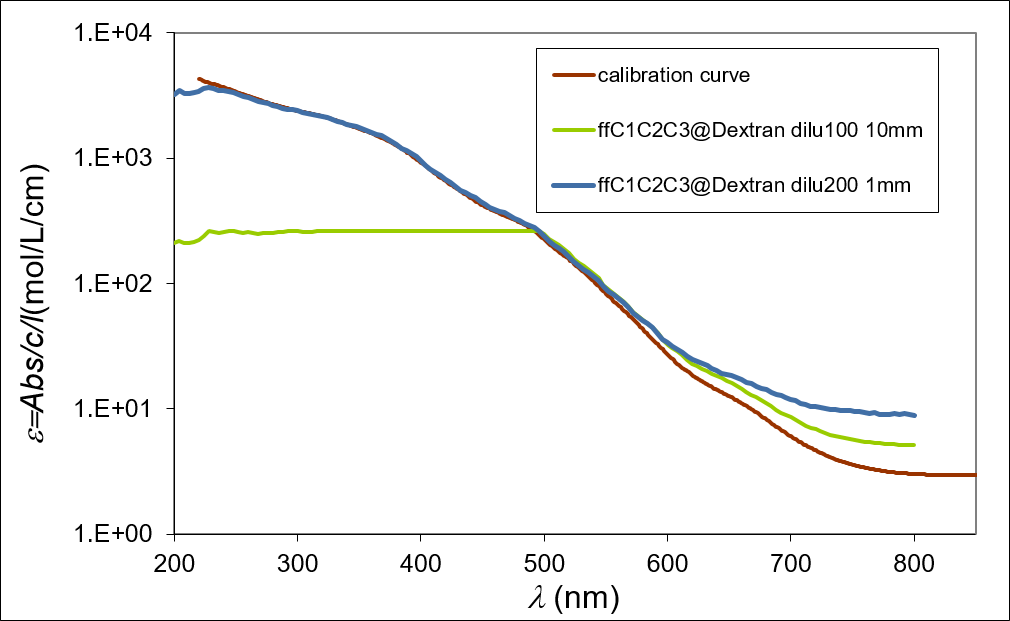


**Figure S3.** UV-vis absorption spectrum (Spectramax™ M2E, Molecular Devices, Sunnyvale, CA, USA) of C1C2C3@dex MNPs diluted 200 times in water for two optical paths (1 and 10 mm).The calibration curve built on atomic emission spectroscopy enables measuring the iron oxide concentration, here 119.8 g/L.


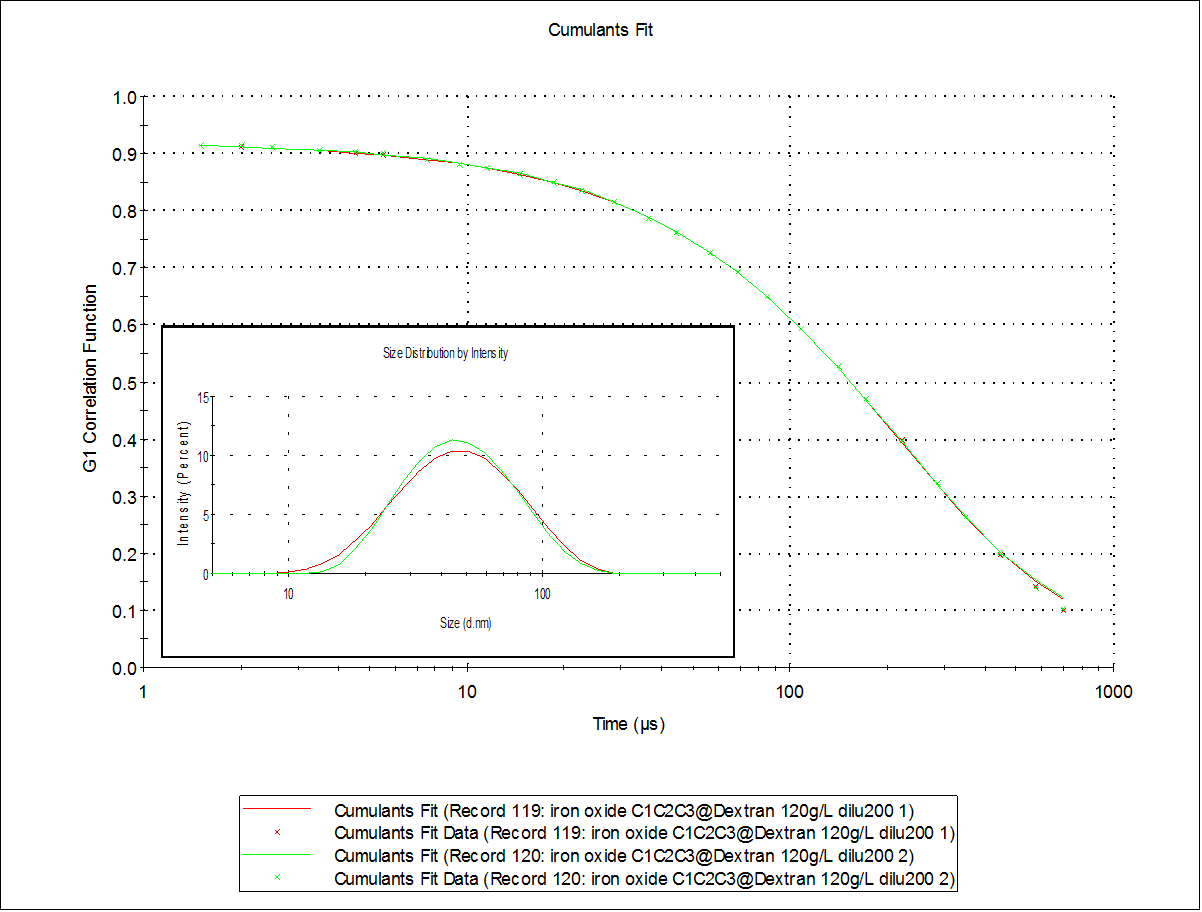


**Figure S4.** Dynamic light scattering (DLS) curve (Nanosizer™, Malvern Instruments, Worcestershire, UK) of C1C2C3@dex MNPs diluted 200 times in water: the fitting of the correlogram by the method of cumulants leads to a Z-average hydrodynamic diameter of 40.2 nm with a polydispersity index PDI = 0.215.


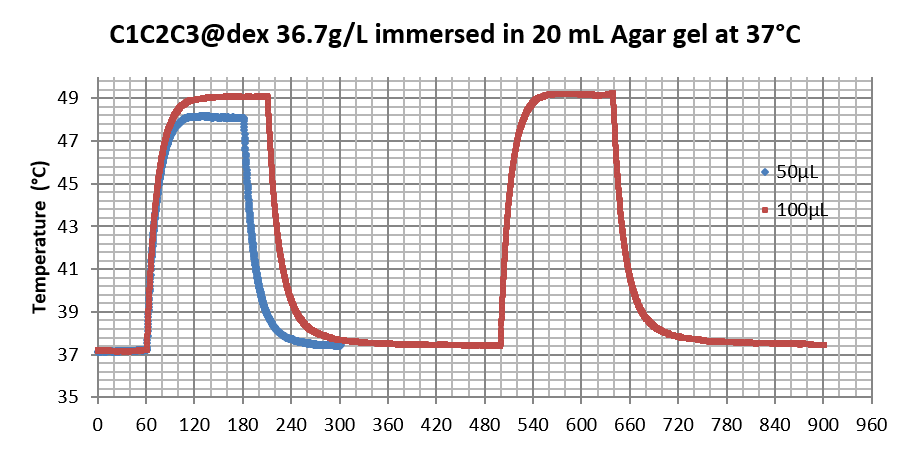


**Figure S5.** Temperature profiles for C1C2C3@dex magnetic nanoparticles (MNPs) in droplets of two volumes (50 and 100 µL) in contact with 20 mL Agar hydrogel maintained at 37 °C by water circulation to mimic the mouse body. The slopes within the first 3 s enable to deduce the specific absorption rates (SAR) of the MNPs: SAR = 92.6 W/g (50 µL) or 95.3 W/g (100 µL).


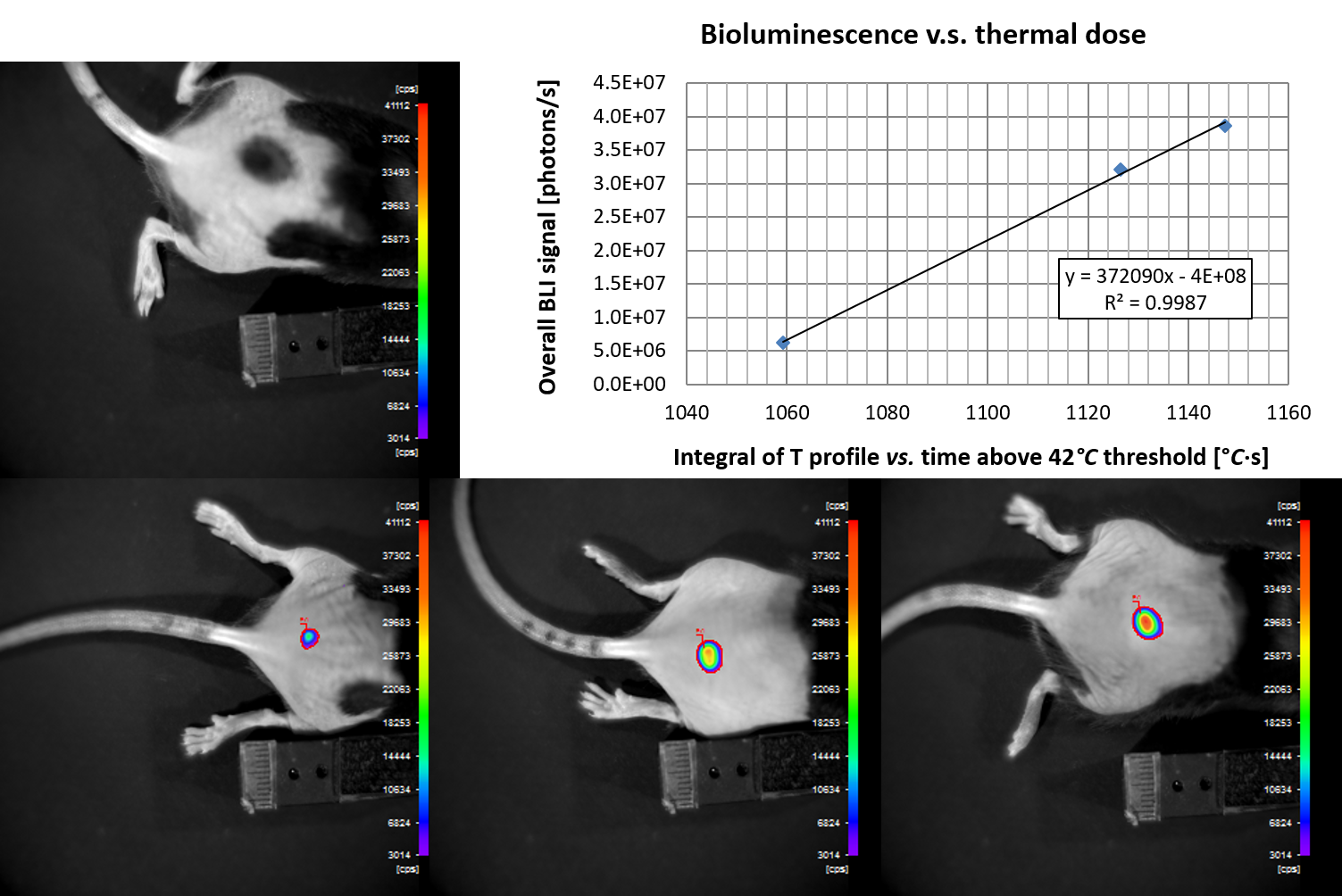


**Figure S6.** Quantitative relationship between thermal dose and BLI signal for topical deposition of MTN droplet on mouse skin.
